# Clinical profile, treatment response, and outcomes in adult primary focal segmental glomerulosclerosis: A single-center experience 

**DOI:** 10.5414/CNP104S11

**Published:** 2025-11-28

**Authors:** Željka Večerić-Haler, Andreja Aleš Rigler, Andrej Škoberne, Špela Borštnar, Nuša Avguštin Rotar, Damjan Kovač, Ana Dovč, Nika Kojc, Jelka Lindič

**Affiliations:** 1 Department of Nephrology, University Medical Center Ljubljana,; 2 Department of Internal Medicine, Faculty of Medicine, and; 3 Institute of Pathology, Faculty of Medicine, University of Ljubljana, Ljubljana, Slovenia

**Keywords:** focal segmental glomerulosclerosis, FSGS, primary FSGS, rituximab, calcineurin inhibitors

## Abstract

Objective: Primary focal segmental glomerulosclerosis (FSGS) is a major cause of nephrotic syndrome in adults. This study evaluates the clinical profile, treatment response, and outcomes of patients with primary FSGS at the University Medical Centre (UMC) Ljubljana, Slovenia. Materials and methods: Patients diagnosed and treated at UMC Ljubljana from 2008 to 2024 were included. Clinical, laboratory, histological, and treatment data were analyzed. Results: 56 patients were followed for a median of 71 months (range: 2 – 186). At diagnosis, 91.1% had nephrotic syndrome, with proteinuria of 10.2 ± 9.8 g/day, serum albumin of 23.7 ± 6.5 g/L, and estimated glomerular filtration (eGFR) of 66.7 ± 27.7 mL/min/1.73m^2^. Histological subtypes included tip (37.5%), cellular (25%), collapsing (14.3%), not-otherwise specified (16.1%), while in 7.1% the variant was not defined. First-line therapy, mainly corticosteroids, led to remission in 67.3%, but 71% experienced relapses or steroid resistance. Calcineurin inhibitors (CNIs) and corticosteroids were used in 20 patients, achieving 60% remission, however the majority of patients experienced relaps following their discontinuation. Rituximab was given to 23 patients with refractory disease, with 78.2% achieving remission (56.5% complete, 21.7% partial) in 5.1 ± 4.9 months. Among initial rituximab responders, 30.4% experienced relapse. At the last follow-up, mean eGFR was 59.6 ± 33.2 mL/min/1.73m^2^, 21.4% progressed to end-stage renal disease (ESRD), and 5.3% died. Four patients underwent kidney transplantation, with early recurrence in 3. Conclusion: Despite high steroid resistance, use of CNIs and rituximab improved remission rates. However, ESRD remains a significant concern, highlighting the need for early intervention and optimized treatment strategies.

## Introduction 

Focal segmental glomerulosclerosis (FSGS) is a common cause of nephrotic syndrome in adults, characterized by focal and segmental scarring of the glomeruli [1, 2]. As a heterogeneous disease, FSGS encompasses primary, secondary, genetic, and adaptive forms, with primary FSGS being attributed to a circulating permeability factor leading to podocyte injury and subsequent proteinuria [3]. The clinical course of primary FSGS is variable, ranging from spontaneous remission to rapid progression to end-stage renal disease (ESRD) [4]. 

Immunosuppressive therapy forms the cornerstone of treatment for primary FSGS, with corticosteroids (CS) and calcineurin inhibitors (CNIs) often serving as first-line agents [5, 6, 7, 8, 9, 10]. However, the response to immunosuppression remains inconsistent, influenced by factors such as the extent of proteinuria, kidney function at presentation, and histopathological variants. While different therapies have shown efficacy in inducing remission in a subset of patients, there remains a significant group that is resistant to conventional treatments. In patients with steroid- or CNI-resistant FSGS, rituximab, a monoclonal antibody targeting CD20-positive B cells, has emerged as a promising therapeutic option [7, 11, 12, 13, 14]. 

Here we present the clinical profile, response to immunosuppressive therapy, and outcomes of adult patients with primary FSGS treated at University Medical Center (UMC) Ljubljana, Slovenia, over a 15-year period. By analyzing data from 56 patients, this work aims to contribute to the growing body of evidence on FSGS and to identify potential predictors of treatment response and disease progression. 

## Materials and methods 

### Patient selection 

We retrospectively analyzed the medical records of all patients diagnosed with primary FSGS who were treated between January 2008 and September 2024 at the Department of Nephrology, UMC Ljubljana. A comprehensive review of medical records initially identified 95 patients with a diagnosis of FSGS. After careful examination of patient documentation and kidney biopsy results, individuals with secondary forms of FSGS were excluded. Ultimately, 56 patients with primary FSGS were selected for further analysis. 

Data on laboratory results, clinical presentation, histological variants, treatment types, and treatment outcomes were extracted from the patients’ electronic medical records. 

The study was approved by the Republic of Slovenia National Medical Ethics Committee (approval number 0120-560/2024-2711-3). 

### Demographic and clinical parameters 

The demographic and clinical parameters analyzed included age at diagnosis, gender, presence of nephrotic syndrome, estimated glomerular filtration rate (eGFR) calculated using the Chronic Kidney Disease Epidemiology Collaboration equation-based on serum creatinine, serum albumin levels, total cholesterol, proteinuria, measured as both 24-hour urinary protein excretion and estimated daily proteinuria (eDP) (urine protein-to-creatinine ratio multiplied with estimated daily excretion of creatinine – 8.8 mmol/day) for follow-up when 24-hour measurements were unavailable. 

Nephrotic syndrome was defined as 24-hour proteinuria > 3.0 g/day or 3.5 g/day/1.73m^2^, hypoalbuminemia (serum albumin ≤ 35 g/dL), generalized edema, and hypercholesterolemia. Nephrotic range proteinuria was defined as 24-hour proteinuria > 3.0 g/day or 3.5 g/day/1.73m^2^ or eDP > 3.5 g/day/1.73m^2^. 

### Histological and treatment data 

Histological analysis included the classification of pathohistological variants (tip, perihilar, cellular, collapsing, and not-otherwise specified (NOS)) according to the Columbia classification and diffuse podocyte effacement by electron microscopy as assessed at the time of treatment [1]. All patients were treated with renin-angiotensin system (RAS) blockers, although this therapy was not analyzed in detail. 

The use of immunosuppressive medications, including CS, CNIs, and other immunosuppressants, was recorded. Adverse effects of immunosuppressive therapy were noted when documented in the medical records. 

### Follow-up and outcomes 

Patients were followed until the last outpatient examination, initiation of dialysis, kidney transplantation, death, or loss to follow-up. ESRD was defined as the initiation of dialysis or kidney transplantation. 

We evaluated response to therapy as:

- Complete remission (complete response to treatment) defined as a reduction in proteinuria to eDP < 0.3 g/day/1.73m^2^;
- Partial remission (partial response to treatment) defined as a reduction in proteinuria by ≥ 50% and below 3.0 g/day or 3.5 g/day/1.73m^2^;
- Relapse defined as re-increase in proteinuria to > 3.0 g/day or ≥ 3.5 g/day/1.73m^2^ in a patient who was in partial or complete remission;
- Steroid dependence defined as a relapse during steroid treatment or 2 weeks after steroid withdrawal or the inability to withdraw CS in order to maintain remission;
- Steroid resistance as an insufficient (without partial remission achieved) or absent reduction in proteinuria after 12 – 16 weeks of treatment with an appropriate, full dose of CS.

### Statistical analyses 

Data analysis was performed using Statgraphics Centurion, version 19. Continuous variables with a normal distribution were expressed as mean ± standard deviation (SD) and compared using Student’s t-test for two-group comparisons or one-way ANOVA for multiple-group comparisons. For continuous variables that did not follow a normal distribution, data were presented as median and interquartile range (IQR) and analyzed using the Mann–Whitney U test. Post hoc analysis was performed for multiple comparisons when ANOVA was significant. Categorical variables were compared using the χ^2^-test. 

Changes in eGFR over time were assessed for the whole cohort and across FSGS variants. Student’s t-test was used to evaluate differences between FSGS variants at specific time points. 

The risk of progression to ESRD in association with FSGS variants was estimated using the Cox proportional hazards model. Hazard ratios (HRs) with 95% confidence intervals (CIs) were reported to assess the impact of FSGS variants on renal survival, with event-free survival defined as the time from renal biopsy to initiation of dialysis, kidney transplantation, or last follow-up. All statistical tests were two-tailed, and a p-value < 0.05 was considered statistically significant. 

## Results 

### Basic and demographic data 

This retrospective study included 56 patients (31 men and 25 women) with a median follow-up period of 71 months (range: 2 – 186 months). At diagnosis, 51 patients (91.1%) presented with nephrotic syndrome, while 5 patients exhibited proteinuria in the nephrotic range. The baseline clinical parameters were as follows: 24-hour proteinuria was 10.2 ± 9.8 g/day, serum albumin 23.7 ± 6.5 g/L, serum cholesterol 8.7 ± 2.7 mmol/L, and eGFR 66.7 ± 27.7 mL/min/1.73m^2^. 

Electron microscopy revealed diffuse foot process effacement in all patients. Histopathological subtypes were identified as follows: 21 patients (37.5%) had the tip variant, 14 patients (25%) had the cellular variant, 9 patients (16.1%) had the NOS variant, and 8 patients (14.3%) had the collapsing variant. The perihilar variant was not observed in any patient. In 4 patients (7.1%), the histopathological variant was not specified at the time of biopsy. 

### Response to first-line therapy 

**Figure 1:**
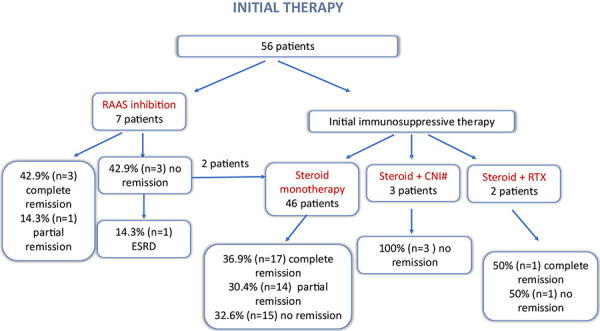
Response to first-line therapy in the UMC Ljubljana cohort of patients with primary focal segmental glomerulosclerosis. RTX = rituximab; CNI = calcineurin inhibitors; ESRD = end-stage renal disease; RAAS = renin angiotensin aldosterone. ^#^All cyclosporine.

The response to first-line therapy is presented in Figure 1. Within the first 3 months post diagnosis, 7 patients received renoprotective treatment without immunosuppressants. In 5 cases, CS therapy was not initiated due to medical or personal reasons, including advanced age, significant kidney injury, or comorbidities. Two patients initially treated with renoprotective therapy – one due to personal reluctance to take CS and another with isolated nephrotic proteinuria – were later started on CS (methylprednisolone at 0.8 mg/kg body weight (BW) daily) and exhibited a steroid-dependent response. One of these patients, who was also less compliant, was lost to follow-up 27 months after diagnosis. 

Among the remaining patients, 44 were treated with CS as monotherapy: 3 received methylprednisolone at 1.6 mg/kg BW on alternate days, 40 patients received methylprednisolone at 0.8 mg/kg BW daily, and 1 patient prednisolone at 1 mg/kg BW. Additionally, 3 patients were treated with cyclosporine combined with a reduced CS dose, and 2 patients received a concomitant combination of methylprednisolone (0.8 mg/kg BW daily) and rituximab. 

Following first-line corticosteroid therapy, 17 patients (36.9%) achieved complete remission, 14 patients (30.4%) achieved partial remission, and 15 patients (32.6%) showed no improvement in 12 – 16 weeks of optimal corticosteroid dose. None of the patients treated with cyclosporine and CS as first-line therapy achieved remission. Among those treated with rituximab and CS, 1 patient achieved complete remission 3 months post treatment initiation, while the other experienced improvement, characterized by the resolution of nephrotic syndrome and reduction in proteinuria; however, partial remission was not achieved within the 6-month observation period. Among steroid responders, the average time to achieve remission was 3.18 ± 1.95 months. 

### Treatment of steroid-dependent and steroid-resistant disease 

A total of 40 patients (71%) who had received first-line immunosuppressive therapy experienced additional relapses or developed steroid resistance. Notably, 30 of them met the formal criteria for either steroid resistance (n = 19) or steroid dependence (n = 11). Retrospective analysis of treatment response showed that 13 steroid-resistant patients exhibited resistance not only to CS but also to multiple immunosuppressive agents, including cyclosporine, tacrolimus, or mycophenolate mofetil. Patients with steroid-dependent disease had a median of 2 relapses (range: 2 – 12), with 4 patients experiencing multiple relapses, defined as more than 3 during the observation period. 

In our cohort, second-line therapy for steroid-dependent and steroid-resistant patients included repeated high-dose CS, CNIs, mycophenolate mofetil, rituximab, or combinations of these treatments. 

Ten patients with relapsing disease were treated repeatedly with full-dose CS monotherapy and achieved remission, while the majority of patients with relapsing or resistant disease received the next level of therapy. Namely, 20 patients were treated with CNIs and CS: 18 were treated with cyclosporine, which was intermittently switched to tacrolimus in 4 cases. Treatment with CNIs resulted in complete and partial remission in 60%, while in 8 patients it was unsuccessful. A total of 11 patients who achieved remission with CNIs (92%) experienced relapse following their discontinuation. Two patients were treated with mycophenolate mofetil. In both, however, the disease progressed to ESRD. 

A total of 23 patients with steroid-dependent (n = 10) and steroid- or CNI-resistant (n = 13) primary FSGS received rituximab, either as adjunct therapy in resistant disease or to reduce relapse frequency in steroid-dependent cases. At the time of rituximab initiation, all patients were on CS or CNIs; however, treatment had either failed to achieve complete remission or was insufficient to prevent multiple relapses. The clinical characteristics and outcomes of patients with steroid-dependent and -resistant FSGS treated with rituximab are summarized in Table 1. These patients exhibited significant disease severity, with an average serum albumin of 23.3 ± 5.36 g/L, eDP of 9.94 ± 5.64 g/day/1.73m^2^, total cholesterol of 8.83 ± 2.53 mmol/L, and an eGFR of 72.91 ± 24.14 mL/min/1.73m^2^. Most patients (91.3%) presented with nephrotic syndrome, while 2 were in partial remission at rituximab initiation due to prior high-dose CS therapy. 

**Figure 2:**
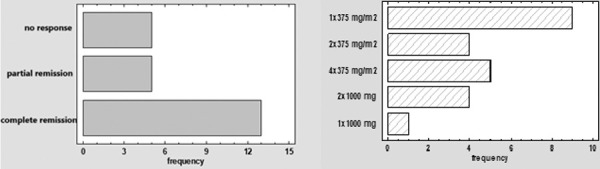
Left: Response to treatment with rituximab. Right: Regimens of rituximab therapy used in patients with focal segmental glomerulosclerosis.

Rituximab was administered using various regimens (Figure 2), including 1 × 375 mg/m^2^ (39.1%), 2 × 375 mg/m^2^ (17.4%), 4 × 375 mg/m^2^ (21.7%), 2 × 1,000 mg (17.4%), and 1 × 1,000 mg (4.4%). The average induction dose was 1,437.95 ± 777.74 mg (range: 620 – 3,200 mg). Patients with higher baseline proteinuria and lower serum albumin levels were more likely to receive higher induction doses (p = 0.04 for both parameters). Despite differences in dosing, no significant association was observed between the dose or regimen and treatment response (p = 0.19 for dose; p = 0.87 for regimen). 

Following rituximab therapy, a total of 13 patients (56.5%) achieved complete remission, 5 (21.7%) partial remission, and 5 (21.7%) showed no response. Among the 10 patients with steroid-dependent disease, all achieved complete remission. Of the patients treated as steroid-resistant (including those marked with ** in Table 1 based on clinical reasoning and treatment limitations) 3 patients (23.1%) achieved complete remission, 5 (38.5%) partial remission, and 5 (38.5%) showed no response. Among responders with steroid- or CNI-resistant disease, the average time to achieve at least partial remission was 5.1 ± 4.9 months (Figure 2). 

Over a mean follow-up of 79.7 ± 46.9 months, 30.4% of initial responders to rituximab experienced a relapse. These patients were re-treated with rituximab, and 4 (17.4%) required rituximab maintenance therapy to sustain remission. Four patients progressed to ESRD, including 2 with tip-variant FSGS, 1 with collapsing FSGS, and 1 with cellular FSGS. 

A significantly higher baseline eGFR (p = 0.02) and lower eDP (p = 0.03) were associated with better treatment response, while a trend toward improved outcomes was observed in patients with multiple relapses (p = 0.08). Response rates did not significantly differ based on FSGS histological variants (p = 0.36), concurrent immunosuppressive therapy at rituximab initiation (p = 0.63), or continuation of prior immunosuppression after rituximab treatment (p = 0.39). Following rituximab therapy, 11 patients (78.6%) continued CNIs until remission was achieved. Notably, those who remained on CNIs post rituximab had significantly higher eDP at initiation (p = 0.009). 

### Outcome of kidney function 

Kidney function progressively declined during the observation period, with distinct trends observed across different FSGS variants at specific time points (initial observed value at kidney biopsy, after 3 months, after 1 year, and at the end of follow-up). The mean eGFR for all patients with FSGS at kidney biopsy, 3 months post diagnosis, 1 year post diagnosis, and at the last follow-up was 66.41 ± 25.99, 59.6 ± 33.20, 69.19 ± 26.79, and 59.66 ± 33.17 mL/min/1.73m^2^, respectively. 

**Figure 3:**
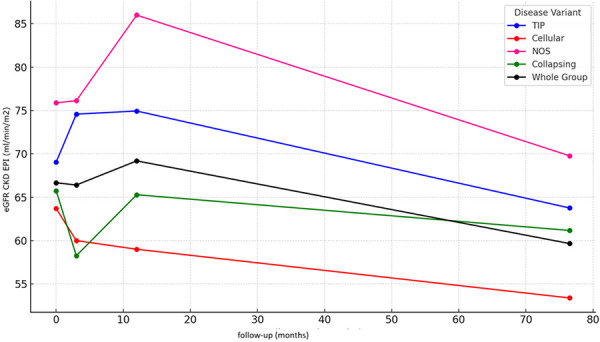
Changes in estimated glomerular filtration rate (eGFR) over time across focal segmental glomerulosclerosis variants. Time points include baseline (at the time of kidney biopsy), 3 months post diagnosis, 1 year post diagnosis, and the end of follow-up. Statistically significant differences in eGFR were observed between variant groups vs. whole group at all time points (p < 0.001 at all time points). Detailed values of eGFR (CKD-EPI, mean ± SD) at each time point, along with statistically significant inter-variant differences, are provided in Supplemental Table 2.

The cellular and collapsing variant demonstrated an initial rapid decrease in eGFR up to 1 year, followed by a subsequent slower decline. In contrast, other variants generally exhibited an initial increase or stable function followed by continuous decrease in eGFR over time. Significant differences in kidney function were observed among FSGS variants at all time points, with the cellular and collapsing variants consistently showing worse kidney function (Figure 3). 

For additional details on mean eGFR, as well as eDP, albumin, and cholesterol levels throughout the follow-up period (median 71 months, range 2 – 186 months), refer to Supplemental Table 1. 

The percentage of patients developing ESRD varied by FSGS variant, being highest in the collapsing (50.0%) and cellular variant (28.6%), followed by the NOS (11.1%) and tip variant (9.5%). A trend was observed between the tip and collapsing variants which was not statistically significant (p = 0.05), and no significant differences were noted between other variants. 

**Figure 4:**
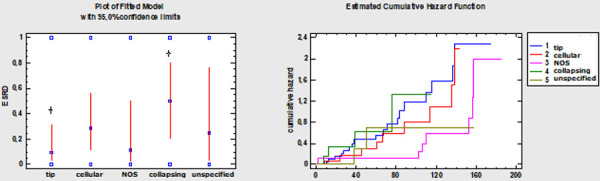
Left: The probability of end-stage renal disease (ESRD) by disease variant (logistic regression, p = 0.05 for tip and collapsing focal segmental glomerulosclerosis (FSGS) at the 95% confidence level). Right: The risk of ESRD in different variants of FSGS in the follow-up time (Cox proportional hazards regression; χ^2^ = 4.24, p = 0.37 at the 95% confidence level).

Although the percentage of patients developing ESRD was highest in the collapsing variant, survival analysis adjusting for time-to-event data (Figure 4) revealed no statistically significant differences in ESRD development between the FSGS variants during the follow-up period. 

During the follow-up period, a total of 12 patients (21.4%) progressed to ESRD, requiring renal replacement therapy. Of these, 4 patients underwent cadaveric renal transplantation, with 3 experiencing early disease recurrence. 

Up to the end of follow-up, 3 patients (5.3%) died. The first fatality was in a 79-year-old woman treated with CS, cyclosporine, and rituximab, who died from acute myocardial infarction 2 months after diagnosis. The second was a 71-year-old man treated with CS and rituximab, who succumbed to gastrointestinal bleeding 9 months post diagnosis. The third was a 69-year-old man treated with CNIs, CS, and rituximab, who passed away from B-cell lymphoma 6 years after completing treatment for FSGS. 

### Adverse effects associated with immunosuppressive therapy 

Based on hospital and outpatient records, 26 patients (46.4%) reported or were treated for complications related to immunosuppressive therapy. Among these, 24 patients experienced adverse effects following the initiation of CS treatment (Supplemental Table 3). The most commonly reported CS-related side effects included: musculoskeletal pain (n = 7), secondary Cushing’s syndrome (n = 7), steroid-induced diabetes (n = 4), infections requiring hospitalization (n = 3), and psychotic disorder (n = 2). 

Two patients previously treated with CS experienced significant complications after rituximab therapy. Both patients had severe nephrotic syndrome caused by collapsing FSGS. The first patient, a 41-year-old man, developed hypogammaglobulinemia, cryptococcal sepsis, and pneumonia, followed by streptococcal sepsis after a dental extraction. The second patient, a 20-year-old woman, suffered from norovirus-induced diarrhea, a metapneumovirus respiratory infection, urticaria-like exanthema (possibly drug- or infection-induced), and severe worsening of hypogammaglobulinemia. 

## Discussion 

The presented study offers valuable insights into the clinical presentation, therapeutic response, and outcomes of 56 adult patients with primary FSGS treated at a single center, with a median follow-up of ~ 6 years. The findings underscore the heterogeneity of FSGS in therapeutic responses, clinical outcomes across histological variants, and reported therapy-associated adverse effects. 

A significant majority of patients presented with nephrotic syndrome, affirming its high prevalence in primary FSGS [15]. Histological variants demonstrated notable diversity, with the tip variant being the most common (37.5%), followed by cellular, NOS, and collapsing variant. These frequencies align with global data on pathologic variants in primary FSGS, as described in D’Agati et al.’s [1] classification system. 

Progression to ESRD occurred in 21.4% of our patients, with the highest percentage observed in the collapsing (50%) and cellular (28.6%) variants, which presented with the lowest eGFR at disease onset. However, after adjusting for time-to-event data, no statistically significant differences in ESRD development were found between the variants. Our findings suggest that, despite existing evidence linking collapsing and cellular variants to the most unfavorable outcomes [15], all variants of FSGS are likely to be aggressive and associated with poorer renal outcomes, particularly when the initial eGFR is severely reduced, underscoring the critical need for a personalized approach to management and treatment. 

Initial immunosuppressive therapy, primarily steroid monotherapy, achieved remission in 67.3% of cases, with 36.9% achieving complete remission. However, the high prevalence of steroid-dependent and steroid-resistant disease (54%) shows the limitations of CS as a standalone treatment. Additionally, the substantial burden of steroid-related adverse effects – such as secondary Cushing’s syndrome, diabetes, musculoskeletal pain, and psychiatric disorder observed in our cohort further constrains their long-term utility [5, 16]. 

CNIs achieved partial or complete remission in 60% of patients with steroid-resistant or relapsing disease, demonstrating their efficacy as a second-line therapy [17, 18]. Nevertheless, the 40% treatment failure rate, the high incidence of relapse following CNI withdrawal, and the progression to ESRD in severe histological subtypes, such as collapsing FSGS, underscore the need for alternative therapeutic strategies. 

Rituximab was administered to 23 patients with steroid-dependent or steroid-(multidrug)-resistant FSGS, achieving an overall remission rate (complete or partial) of 78.2%. In steroid-dependent patients, rituximab effectively induced remission in 100% of cases, while in steroid-resistant disease, at least partial remission was achieved in 54% of patients. This high efficacy, even in refractory cases, highlights the reasoning behind rituximab’s growing role in the management of FSGS [11, 12, 13, 14, 19, 20, 21]. Notably, our study demonstrated higher remission rates with rituximab in both steroid-resistant and -dependent FSGS compared to other reports [12, 22]. 

A key factor that may explain this enhanced response, particularly in severely affected patients with the highest proteinuria, is our consistent approach of continuing CNIs alongside rituximab, rather than discontinuing them after rituximab initiation. This therapeutic strategy likely enhanced treatment outcomes observed in our cohort. Namely, more then 70% of patients continued concomitant CNIs until remission which suggests that rituximab alone could be insufficient for complete disease control in the most severe cases. This finding aligns with existing literature, where CNIs are often required as adjunct therapy to maximize podocyte protection [10]. 

Interestingly, response rates in our study were independent of rituximab dose or regimen, aligning with findings from Hansrivijit et al. [12], who reported no significant differences in remission or relapse rates between low-dose (< 1,500 mg/m^2^) and higher-dose protocols. These results support the hypothesis that rituximab’s efficacy may stem from stabilizing the podocyte cytoskeleton, in addition to its immunomodulatory effects, which appear to be dose-independent [20]. Notably, we observed a strong correlation between lower baseline proteinuria and better response to rituximab, suggesting that disease severity at the time of treatment initiation may play a crucial role in therapeutic success. Although rituximab dosing in this cohort did not appear to be tailored to baseline proteinuria severity, we suggest that patients with very high proteinuria might benefit from alternative dosing strategies, such as combination therapy with CNIs. Furthermore, better outcomes were observed in patients with relapsing disease and higher baseline eGFR, reinforcing the idea that early rituximab initiation may help preserve renal function [12]. Future studies should explore whether tailored rituximab dosing based on proteinuria burden could optimize treatment efficacy in FSGS. 

The observed relapse rate in our cohort (30.4%) is in line with previous studies on rituximab in nephrotic syndrome, suggesting that while rituximab is effective in inducing remission, it does not guarantee long-term disease control [13, 14, 23]. Maintenance rituximab therapy was administered to a small subset of our patients (17.4%) who experienced frequent relapses after achieving rituximab-induced remission or attained partial remission in the setting of severe, multidrug-resistant nephrotic syndrome. However, the role of rituximab in FSGS maintenance therapy remains an area of ongoing research. 

Despite the above stated benefits of rituximab therapy, severe cases like collapsing FSGS showed mixed outcomes with rituximab, mirroring observations in other studies [21]. Moreover, 2 patients with collapsing FSGS developed severe infections and hypogammaglobulinemia following rituximab therapy, emphasizing the need for careful monitoring and further research to optimize its use in severe nephrotic syndrome. 

While rituximab has a higher upfront cost, its superior remission rates and reduced adverse effects offer a possible cost-benefit advantage. This is particularly evident in steroid-resistant and relapsing cases, in patients suffering significant steroid-related adverse effects, and in those at high risk of ESRD. Delaying progression in these patients could substantially reduce long-term healthcare costs, reinforcing the rationale for its use in FSGS. 

## Conclusion 

Management of primary FSGS in adults is challenging due to significant variability in clinical presentation, treatment response, and renal outcomes. While first-line steroids are effective for some, many patients require additional therapies, including CNIs, rituximab, or a combination of both in severe or refractory cases. 

Our findings underscore the high efficacy of rituximab in steroid-resistant and relapsing FSGS, particularly when combined with CNIs, highlighting the need for a tailored, multimodal treatment approach. The sustained remission rates observed suggest that integrating rituximab into individualized regimens can improve long-term disease control while minimizing prolonged steroid exposure. 

Further research is needed to optimize rituximab dosing, identify predictors of response, and refine combination strategies to enhance patient outcomes and reduce healthcare costs. 

## Authors’ contributions 

Conceptualization, Ž.V.H.; methodology, Ž.V.H.; acquisition of patient data, Ž.V.H. and N.K.; formal analysis, Ž.V.H.; validation, A.Š.; writing – original draft preparation, Ž.V.H.; writing – review and editing, J.L., A.Š., A.A.R., Š.B., N.A.R., D.K., A.D.; visualization, Ž.V.H., A.Š. All authors read and approved the final version of the manuscript. 

## Funding 

This work did not receive any funding from public, commercial, or non-profit organizations. 

## Conflict of interest 

Authors declare no conflict of interests. 

[Table SupplementalTable1], [Table SupplementalTable2], [Table SupplementalTable3]

**Table 1.** Clinical features and outcomes of patients with steroid-dependent and steroid-(or multidrug)-resistant focal segmental glomerulosclerosis treated with rituximab. All data are expressed as average ± SD or median and interquartile range.

Id	Age	Histology type	Nephrotic syndrome	Albumin (g/L)	Daily proteinuria (g)	Egfr ckd epi (mL/min/1.73m^2^)	Cholesterol (mmol/L)	Definition of steroid response	Therapy prior to rituximab	Rituximab regimen	Therapy following rituximab	Outcome
1	68	Tip	Yes	29	8.9	82	6.3	**	Tac + low dose steroid	2 × 1,000 mg	None	Partial remission
2	28	Collapsing	Yes	23	11.3	90	10.3	SD	Tac + low dose steroid	2 × 375 mg/m^2^	Tac, steroid ex	Complete remission
3	22	Tip	Yes	nd	6.2	90	8.5	SR	CsA	4 × 375 mg/m^2^	CsA	Complete remission
4	57	Tip	Yes	25	15.0	55	5.6	SR	CsA	1 × 375 mg/m^2^	CsA	Complete remission
5	34	Collapsing	Yes	33	5.7	90	9.6	SR	Tac + low dose steroid	2 × 375 mg/m^2^	Tac + low dose steroid	*No effect, ESRD 116 months post diagnosis
6	81	Tip	Yes	17	11.5	13	7.8	SR	Steroid in full dose + CsA	1 × 375 mg/m^2^	Tac + low dose steroid	No effect-ESRD 26 months post diagnosis
7	46	NOS	No	34	2.6	90	nd	SD	Steroid in full dose	4 × 375 mg/m^2^	Tac, steroid ex	Complete remission
8	39	Tip	No	30	2.5	71	nd	SD	Steroid in full dose	1 × 375 mg/m^2^	None	Complete remission
9	45	NOS	Yes	21	11.3	90	9.3	SD	Steroid in full dose + CsA	1 × 1,000 mg	Low dose steroid	Complete remission
10	26	Tip	Yes	24	14.0	73	nd	SR	Steroid in full dose + CsA	4 × 375 mg/m^2^	CsA	Partial remission
11	43	NOS	Yes	25	3.9	90	6.4	SD	Steroid in full dose	1 × 375 mg/m^2^	None	Complete remission
12	66	Cellular	Yes	17	7.1	46	8.9	**	Steroid in full dose + CsA	1 × 375 mg/m^2^	None	No effect, ESRD 37 months post diagnosis
13	51	Cellular	Yes	20	6.3	62	8.1	SD	Steroid in full dose	2 × 1000 mg	None	Complete remission
14	18	Collapsing	Yes	16	9.0	62	9.0	SR	Steroid in full dose + CsA	4 × 375 mg/m^2^	CsA, rituximab maintenance	Partial remission
15	24	Cellular	Yes	22	13.0	122	9.3	SD	CsA + low dose steroid	1 × 375 mg/m^2^	CsA, rituximab maintenance	Complete remission
16	69	Tip	Yes	20	9.8	82	8.1	**	CsA + low dose steroid	1 × 375 mg/m^2^	CsA	Complete remission
17	41	Collapsing	Yes	23	5.6	90	10.5	SR	Steroid in full dose, MPF, MMF	1 × 375 mg/m^2^	CsA	Partial remission
18	33	Tip	Yes	20	25.9	83	10.5	SR	Steroid in full dose + Tac	2 × 375 mg/m^2^	IVIg (low IgG)	*No effect, ESRD 78 months post diagnosis
19	37	NOS	Yes	26	9.1	80	9.3	SD	Steroid in full dose + CsA	2 × 375 mg/m^2^	None	Complete remission
20	63	Not determined	Yes	27	8.3	28	4.9	SR	Steroid in full dose	2 × 1,000 mg	Rituximab maintenance	No effect, progressive decline of kidney function
21	20	Collapsing	Yes	14	12.0	52	15.0	SR	Steroid in full dose	4 × 375 mg/m^2^	CsA	Partial remission
22	45	Cellular	Yes	19	19.9	61	11.3	SD	Steroid in full dose + CsA	2 × 1,000 mg	CsA, rituximab maintenance	Complete remission
23	75	Tip	Yes	27	9.6	75	7.9	SD	Steroid in full dose	1 × 375 mg/m^2^	None	Complete remission

NOS = not otherwise specified; eGFR = estimated glomerular filtration rate; CsA = cyclosporine; IVIg = intravenous immunoglobulin; Tac = tacrolimus; MMF = mycophenolate mofetil; MPF = membrane plasmapheresis; SR = steroid-resistant; SD = steroid-dependent; ESRD = end-stage renal disease; nd = no data. *Patient underwent kidney transplantation; **patient #1 was never treated with optimal high dose of steroids due to personal reluctance, patient #12 initially responded to steroids but had to discontinue treatment due to aseptic bone necrosis, patient #16 achieved complete remission with high-dose steroids but required further treatment following two relapses.

**Supplemental Table 1 SupplementalTable1:** Laboratory results during follow-up in patients with FSGS.

Patients with FSGS n = 56	Age (years)	Follow-up (months)	Albumin (g/)	Cholesterol (mmol/L)	eDP (g/day)	eGFR (mL/min/1.73m^2^)	Albumin 3 months (g/L)	Cholesterol 3 months (mmol/L)	eDP 3 months (g/day)	eGFR 3 months (mL/min/1.73m^2^)	Albumin 1 year (g/L)	Cholesterol 1 year (mmol/L)	eDP 1 year (g/day)	eGFR 1 year (mL/min/1.73m^2^)	Albumin end of follow-up (g/L)	eDP end of follow-up (g/day)	eGF end of follow-up (mL/min/1.73m^2^)
Average	45.9	76.5	23.7	8.73	10.2	66.7	33.57	6.1	5.5	66.4	37.9	5.8	2.8	68.7	39.6	3.1	59.7
Standard deviation	19.1	52.7	6.6	2.68	9.8	27.74	8.1	2.4	8.5	25.9	6.9	1.9	3.0	31.1	7.6	4.3	33.2

eGFR = estimetd glomerular filtration; eDP = estimated daily proteinuria.

**Supplemental Table 2 SupplementalTable2:** Changes in eGFR (CKD-EPI, mean ± SD) over time across different FSGS variants are presented. Time points include baseline (at the time of kidney biopsy), 3 months post-diagnosis, 1 year post-diagnosis, and the end of follow-up. Statistically significant differences in eGFR between FSGS variants were assessed using Student’s t-test (p < 0.05 considered significant) and are indicated in the table as superscript letters.

Variant	eGFR biopsy	eGFR 3 months	eGFR 1 year	eGFR end of follow-up
Tip^1^	70.61 ± 27.86^2,3,4^	74.58 ± 19.25^2,4^	74.11 ± 25.83^2,4^	67.89 ± 27.88^2^
Cellular^2^	61.50 ± 26.48^1^	60.00 ± 24.69^1,3^	59.00 ± 22.57^1,3^	50.33 ± 33.59^1,3^
NOS^3^	82.86 ± 11.46^1,4^	76.12 ± 23.74^2,4^	86.00 ± 4.41^1,2,4^	75.57 ± 26.19^2,4^
Collapsing^4^	59.80 ± 28.29^1,3^	58.25 ± 28.95^1,3^	60.40 ± 37.38^1,3^	60.40 ± 37.16^3^

^1^Confirmed statistical differences with the tip category; ^2^Confirmed statistical differences with the cellular category; ^3^Confirmed statistical differences with the NOS category; ^4^Confirmed statistical differences with the collapsing category.

**Supplemental Table 3 SupplementalTable3:** Reported adverse effects after corticosteroid therapy in patients with FSGS.

Patient	Reported side effects after corticosteroid therapy
1	Muscle pain
2	Osteomuscular pain, paresthesias, secondary Cushing’s syndrome
3	Hemorrhoids, bruises, secondary Cushing’s syndrome
4	Multiple bacterial infections (that required hospitalisation), steroid diabetes, psychotic disorder
5	Severe leucocytosis
6	Urosepsis, CMV colitis, intestinal bleeding, acute miocardial infarction
7	Secondary Cushing’s syndrome, osteopenia, hirsutism, weight gain
8	Many psychiatric hospitalizations due to exacerbation of psychosis
9	Worsening of arterial hypertension
10	Myopathies, secondary Cushing’s syndrome
11	Secondary Cushing’s syndrome
12	Cataract, steroid diabetes
13	Steroid diabetes, suffusions
14	Aseptic necrosis of the hip
15	Myalgias, osteopenia
16	Soor esophagitis
17	Severe sacroiliitis
18	Steroid diabetes
19	Stretch marks, secondary Cushing’s syndrome
20	Subscapularis tendon rupture
21	CMV viremia
22	Osteomuscular pain
23	Muscle pain
24	Osteomuscular pain, paresthesias, secondary Cushing’s syndrome
